# Elucidating the Reprograming of Colorectal Cancer Metabolism Using Genome-Scale Metabolic Modeling

**DOI:** 10.3389/fonc.2019.00681

**Published:** 2019-07-30

**Authors:** Cheng Zhang, Mohammed Aldrees, Muhammad Arif, Xiangyu Li, Adil Mardinoglu, Mohammad Azhar Aziz

**Affiliations:** ^1^Science for Life Laboratory, KTH - Royal Institute of Technology, Stockholm, Sweden; ^2^Department of Medical Genomics, King Abdullah International Medical Research Center, Riyadh, Saudi Arabia; ^3^King Saud Bin Abdul Aziz University for Health Sciences, Riyadh, Saudi Arabia; ^4^Ministry of the National Guard- Health Affairs, Riyadh, Saudi Arabia; ^5^Department of Biology and Biological Engineering, Chalmers University of Technology, Gothenburg, Sweden; ^6^Centre for Host–Microbiome Interactions, Dental Institute, King's College London, London, United Kingdom; ^7^Colorectal Cancer Research Program, King Abdullah International Medical Research Center, Riyadh, Saudi Arabia

**Keywords:** colorectal cancer, genome scale metabolic model, polyamine metabolism, personalized medicine, transcriptomics

## Abstract

Colorectal cancer is the third most incidental cancer worldwide, and the response rate of current treatment for colorectal cancer is very low. Genome-scale metabolic models (GEMs) are systems biology platforms, and they had been used to assist researchers in understanding the metabolic alterations in different types of cancer. Here, we reconstructed a generic colorectal cancer GEM by merging 374 personalized GEMs from the Human Pathology Atlas and used it as a platform for systematic investigation of the difference between tumor and normal samples. The reconstructed model revealed the metabolic reprogramming in glutathione as well as the arginine and proline metabolism in response to tumor occurrence. In addition, six genes including ODC1, SMS, SRM, RRM2, SMOX, and SAT1 associated with arginine and proline metabolism were found to be key players in this metabolic alteration. We also investigated these genes in independent colorectal cancer patients and cell lines and found that many of these genes showed elevated level in colorectal cancer and exhibited adverse effect in patients. Therefore, these genes could be promising therapeutic targets for treatment of a specific colon cancer patient group.

## Introduction

Colorectal cancer (CRC) is one of the most common malignant diseases. It has third highest incidence among all cancers worldwide. Due to better screening and early interventions, the mortality of CRC has been decreasing. However, the development of chemotherapy option has seen a marginal improvement. 5-Fluorouracil based chemotherapy is currently the first-line treatment against CRC (1), but the response rate of 5-Fluorouracil is only around 10–15% among patients (2). Several new additions in the form of oxaliplatin, irinotecan, capecitabine, cetuximab, and bevacizumab and other biologicals have become available mostly as combination therapies. The level of success anticipated with the targeted therapeutic agents failed on various parameters (3–7). Therefore, there is still great interest in development of effective treatment strategies for CRC.

While studying colorectal cancer patients locally in Saudi Arabia, we came across the heterogeneity in the somatic cytogenetic and transcriptomic level changes associated with this type of cancer. There were copy number changes that were reported earlier as well as novel events that characterized this patient population (8, 9). The transcription level changes suggest the involvement of different genes in initiation and progression of CRC in these same patients. An integrated analysis with data from cytogenetic and transcriptomic level measurement provided the gene targets that carry higher confidence value than either of them alone (10). The next level in the functional hierarchy is protein and metabolites which are the effector molecules that execute the assigned tasks. Understanding metabolite level changes related to colorectal cancer in an integrated manner would increase the confidence level in finding the biomarkers and therapeutic targets that can be accurate, precise, and clinically useful.

Colon cancer undergoes severe metabolic reprogramming during its transformation and systems biology tools can be employed for identification of the altered metabolic processes. To this end, the reconstruction of genome-scale metabolic models (GEMs) can help to knit high-throughput data (i.e., proteomics and transcriptomics) in the network topology and a variety of methods enable the researchers to predict how these transcriptional perturbations are translated into alterations in distinct and biologically meaningful metabolic subsystems (11–13). GEMs condense information about the known functions of protein-encoding genes, how these genes/proteins interact with other bioactive compounds and associated reactions, allowing robust and reliable analyses to be performed by high-quality bioinformatics pipelines and similar tools (14). These integrative models have been successfully employed to gain further biological and mechanistic understanding of metabolism related disorders (15, 16).

To date, different GEMs for cancer have been reconstructed to characterize the genetic mechanisms of cancer and to reveal how cancer cells benefit from metabolic modifications (17–21). In this report, we reconstructed generic GEM for colon cancer tumors by integrating personalized GEMs that are generated based on the Human Metabolic Reaction 2 (HMR2) database (11, 22) and transcriptomics data (RNA-Seq) in The Cancer Genome Atlas (TCGA). The reconstructed model has been used for the analysis of the gene expression data generated from colon cancer samples and matched normal samples of patients. These analyses revealed the key metabolic alteration around arginine and proline metabolism as well as glutathione metabolism.

## Materials and Methods

### Patient Samples and mRNA Expression Profiling

Patient sample collection and RNA extraction were performed as previously described (10). Briefly, 46 tumor samples and 44 adjacent normal samples were obtained after the requisite approval of Institutional Review Board at King Abdullah International Medical Research Center by King Abdul Aziz Medical City, Riyadh, Saudi Arabia. The samples were obtained from biopsies as well as surgical resections. All the patients signed the Institutional Review Board informed consent form prior to sample collection. All samples were immediately stored in the RNA later reagent until the extraction of nucleic acids. Methods mentioned herein were performed in accordance with the relevant guidelines and regulations. Homogenization was carried out using a QiaPrep homogenizer (Qiagen, Hilden, Germany) with stainless steel beads (5 mm). Total RNA was isolated using the Macherey-Nagel TripPrep kit (Macherey-Nagel Inc., Bethlehem, PA, USA) with <30 mg of tissue. Human Exon ST 1.0 arrays from Affymetrix (Santa Clara, CA, USA), containing probes set for 22,011 genes, were used as previously described together with the amplification and labeling kit from Ambion (Foster City, CA, USA).

All mRNA expression data has been deposited in the GEO database under the accession number GSE50421 and GSE77434 (90 samples: 44 normal samples and 46 cancer samples). Data were exported as CEL files (which contain data on the intensity of each signal, indicating the expression level of the gene corresponding to each probe) and processed using Expression Console software (Affymetrix). All data was subjected to quantile normalization using Integromics Omics office licensed software available at www.integromics.com. Fold change values for metabolic genes were obtained using Transcriptomics analysis console (Affymetrix, Thermofisher scientific, USA).

### Animal Cell Culture

HCT8, HCT 116, SW480, and SW620 colorectal cancer cell lines were obtained from American Type Culture Collection, USA. All cell lines were maintained in 10% Fetal Calf Serum (Gibco) in Dulbecco's modified minimal eagle's media (DMEM) obtained from Thermo Fischer Scientific. Culture conditions were: 5% CO_2_ at 37°C in a humidified incubator. RNA extraction from these cell lines was done using Macherey-Nagel TripPrep kit (Macherey-Nagel Inc., Bethlehem, PA, USA) followed by cDNA conversion using kit (Roche).

### Quantitative Real Time Polymerase Chain Reaction

Gene expression levels for the candidate genes were obtained from matched tumor-normal samples of 15 patients. A Taqman assay from Applied Biosystems (Thermo Fisher Scientific, USA) was used along with the Taqman master mix. 18S rRNA was used as housekeeping gene. All the samples were done in quadruplicate using ABI 7900 instrument. Gene expression analysis software from ABI was used to calculate fold change values using the ΔΔCt method (i.e., RQ = 2^−ΔΔ*Ct*^).

### Gene Set Enrichment Analysis and Bioprofiler Analysis

The KEGG and GO enrichment analysis was done with PIANO (23) package in R. The gene-set collections for GO biological process and KEGG pathways was taken from MSigDB (24). GO and KEGG terms with FDR <5% in distinct-directionality class are considered as significantly changed. Distinct-directionality class in PIANO result is using the proportion between significantly up- and down-regulated genes from differential expression to define the statistical value and significance level. Bioprofiler analysis was carried out to know the causal relationship of the six identified genes with CRC. It was done using Ingenuity pathway analysis software (Qiagen biosciences).

### Reconstruction of GEMs

The generic human colorectal cancer GEM was reconstructed based on unification of the 374 individual GEMs created in Human Pathology Atlas (downloaded from BioModels Database www.ebi.ac.uk/biomodels) to cover the all individual variations among colorectal cancer patients. The reporter metabolite analysis is performed using RAVEN toolbox implemented in Matlab 2017a.

### Kaplan-Meier Analysis

The Kaplan-Meier survival analysis and the log-rank *P*-value is obtained from pathology atlas (21). We only included data from dead subjects for robustness, and the default best separation cutoff for each gene provided by pathology atlas is used to stratify the patients.

## Results

### Transcriptomic Comparison Between Colon Cancer Tumor and Normal Tissue

To investigate the transcriptomic changes in colon cancer tumor, we performed differential expression analysis using microarray data from 46 cancer samples and 44 normal samples. Consequently, we identified 2012 up-regulated genes and 1373 down-regulated (FDR < 0.05; Table S1), respectively. Among up-regulated genes, we found key glycolytic enzymes, PFKM, GPI, PGAM5, PKM, and LDHD, and these indicated an enhanced glycolytic process. This is a strong indication for Warburg effect, which is well-known to be present in colorectal cancer cells. We also found FASN, ACLY, ACACA, and SCD are significantly up-regulated, which indicated an increased fatty acid biosynthesis. It is clinically well-known that fatty acid synthesis is increased in colorectal cancer (25), and this could help the cancer cell to produce lipid metabolites crucial to cell membranes, cell signaling, post-translational modifications of proteins, neutralization of toxic reactive oxygen species, and energy storage (26). In addition, we found GLS2 is also significantly up-regulated, which indicated an increased uptake of glutamine and suggested increased glutaminolysis in the cancer cells.

**Figure 1:**
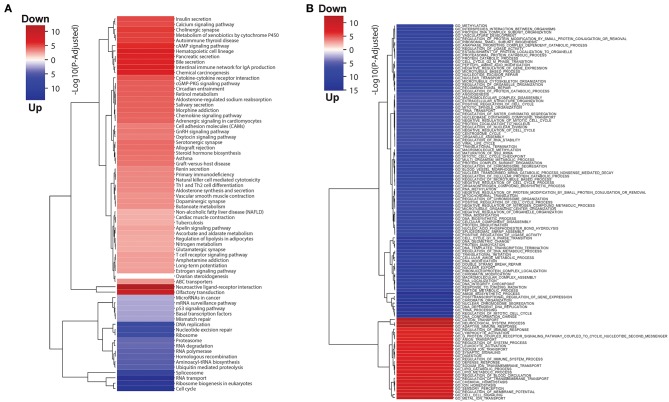
**(A)** KEGG pathway and **(B)** GO terms enrichment analysis results for differentially expressed genes in colon tumor. The blue and red colors represent up- and down-regulated in colon tumor compared to normal samples, and the intensity of the color indicates the minus log *P*-value.

We also performed GO term and KEGG pathway enrichment analysis for these differentially expressed genes (Figure 1; Tables S2, S3). The up-regulated genes were significantly enriched in cell cycle related pathways (cell cycle in KEGG and cell cycle check point in GO), and the down-regulated genes were significantly enriched in the immune related pathways (T cell receptor signaling pathway in KEGG and leukocyte activation in GO), which are expected in cancer and could be treated as good positive controls. In addition, down-regulated genes were also significantly enriched in fatty acid catabolic metabolism pathways in KEGG enrichment analyses, which might be related to normal colon function. Interestingly, we also found several metabolic pathways, for instance oxidative phosphorylation, branch amino acids metabolism, and tricarboxylic acid cycle (TCA) were enriched with down-regulated genes in colon cancer. This motivates further investigation in metabolic shift in colon tumors.

### Identification of Key Metabolic Pathways Using Functional GEMs

In order to study the metabolic reprogramming of the colorectal cancer patients accounting for the differences at the individual level, we integrated the personalized colorectal cancer GEMs and generated a generic GEM for colorectal cancer. This GEM includes 2,628 genes, 4,753 metabolites, and 6,716 biochemical reactions that happen in 8 different subcellular compartments. We compared the model with previously published healthy colon tissue model (27). We found that 467 genes, 681 metabolites, and 1,084 reactions are specific to colon cancer model and it is the largest colorectal cancer GEM.

We employed the GEM of colorectal cancer for the analysis of the RNA-Seq data using indigenously generated microarray data and identified the highly perturbed metabolites and modules between the two conditions using Reporter Metabolite and Reporter Subnetworks algorithms, respectively (28). Reporter Metabolite analysis would allow for the identification of metabolites in the network for which there is significant enrichment of associated gene expression changes and the Reporter Subnetwork algorithm would allow for the identification of a set of metabolic reactions that exhibit transcriptional correlation after a perturbation. These algorithms could provide valuable information on transcriptional regulation on changes in cell conditions and allowed for identification of metabolic reprogramming in colorectal cancer metabolism.

**Figure 2:**
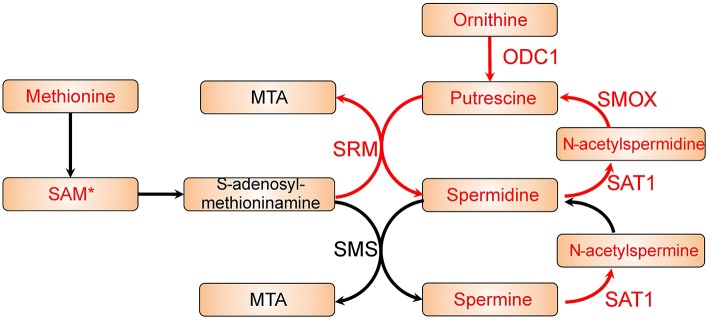
Polyamine metabolic pathway with differential expression genes and reporter metabolites highlighted. Metabolites are shown as bricks and reporter metabolites are highlighted in red. Arrows represent metabolic reactions, and the genes related to the reactions are annotated closely to the arrows. The genes in red are significantly up-regulated in the differential expression analysis, and the reporter metabolites and genes with a star are border line significant (FDR ~ 0.1).

The reporter metabolites have been provided in Table S4, and several polyamines appeared as top significant reporter metabolites. We identified putrescine, spermidine, and N1-acetylspermidine are among the top up-regulated reporter metabolites in colon cancer. These are all polyamines and indicate an increased polyamine metabolic activity in colon cancer cells. Polyamines has reported to be natural anti-oxidants that could protect cells from oxidative damage (29). It has also been reported before that patients with malignant disease has higher urine polyamines level (30). Therefore, polyamines metabolism might play a key role in colon cancer. As shown in Figure 2, by mapping the differentially expressed genes and the reporter metabolites, we observed that the metabolic pathway from ornithine to spermine and spermidine is up-regulated in colon cancer cells. We found most of the key enzymes are significantly up-regulated. For instances, ODC1, SRM, and SMOX are significantly up-regulated, and the up-regulation of SAT1 is border line significant (FDR = 0.1). In addition, another key enzyme, SMS, is appeared in the reporter subnetwork analysis even though it is not annotated in the microarray data. These together suggested that polyamine metabolism is essential in colon cancer.

### Validation of Candidate Genes

**Figure 3:**
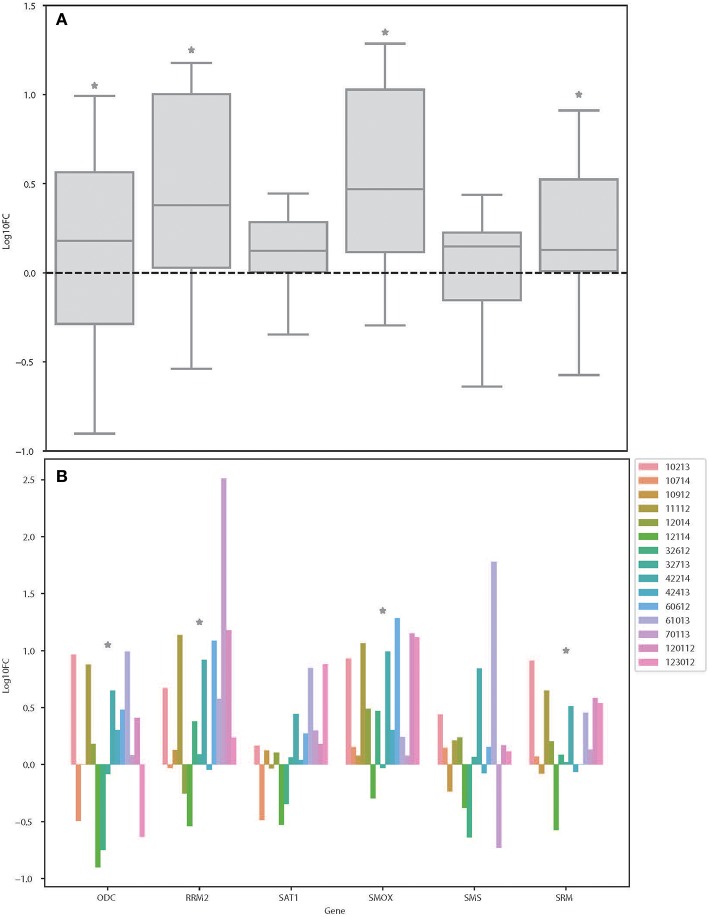
Expression changes of ODC, RRM2, SAT1, SMOX, SMS, and SRM in colorectal cancer patients. **(A)** Boxplot showing the log fold changes of the indicated genes among the patients. **(B)** Bar plot showing the log fold changes of each gene in each patient. ^*^Denotes the genes that is significantly differentially expressed between colon cancer and normal samples as shown in Table S1 (FDR < 0.05).

**Figure 4:**
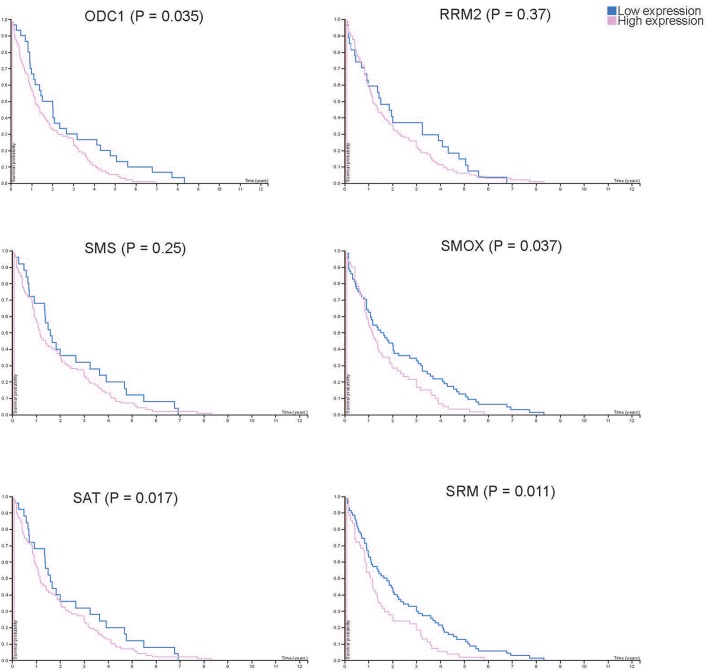
Kaplan-Meier plots showing the survival difference between patient groups with high and low RNA expression of each key gene. Blue and pink lines show the survival of patient group with low and high key gene expression, respectively.

**Figure 5:**
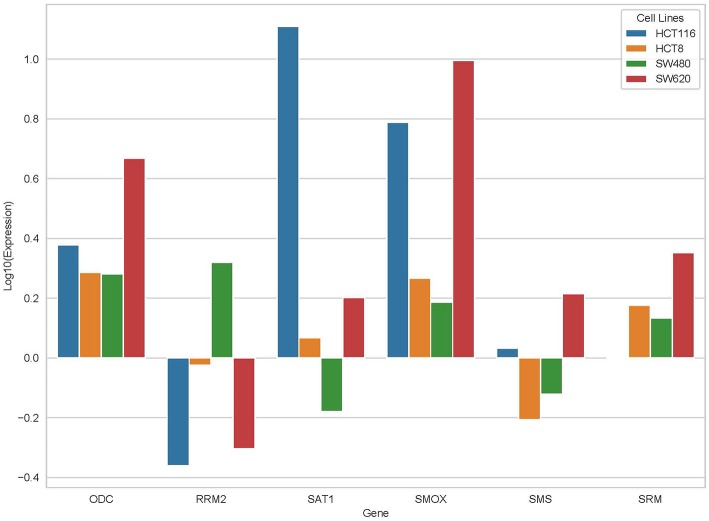
Expression changes of ODC, RRM2, SAT1, SMOX, SMS, and SRM in four cancer cell lines, HCT116, HCT8, SW480, and SW620.

From the results based on the reconstructed functional GEMs, six genes (ODC1, SMS, SRM, RRM2, SMOX, and SAT1) were identified to be playing significant role in causing metabolic changes. The expression level of five of these genes as deduced from patient gene expression microarray data is given in Table 1. For SMS gene we couldn't find the correct annotation in the microarray results. We validated the expression levels of these genes in a subset of 15 patients as well as four colorectal cancer cell lines. While we observed a general pattern exhibiting upregulation of ODC1, RRM2, SAT1, SMOX, SMS, and SRM genes (Figure 3A), heterogeneity among the patient group was conspicuous (Figure 3B). In addition, we also investigated the prognostic roles of these genes in TCGA colorectal cancer cohort and obtained the log-rank *P*-values from Pathology Atlas (21). As shown in Figure 4, we found higher expression of all six genes to be associated with adverse effect on patients. Among the six genes, the adverse effects of ODC1, SRM, SMOX, and SAT1 exhibited statistical significance (log-rank *P* < 0.05). This further confirms their key roles in colorectal cancer. As shown in Figure 5, in confirmation with patient sample data, these six genes showed similar expression changes in cell lines. For instance, the prognostic genes ODC1 and SMOX showed upregulation in HCT8, HCT116, SW480, and SW620 cell lines. In addition, SRM was up-regulated in three cell lines, and stayed unchanged in HCT116. SAT1 exhibited downregulation only in SW480 cell line. On the other hand, SMS and RRM2 exhibited heterogeneous expression pattern in cancer cell lines, which is in agreement to the survival analysis results. All values of gene expression fold changes in patients as well as cell lines are given in supplementary data Table S5. In summary, the roles of ODC1, SMOX, SRM, and SAT seems more important and conserved according to both *in vivo* and *in vitro* analysis.

**Table 1:** Fold change values for metabolic genes in patient tumor samples.

**Gene symbol**	**Description**	**Fold change**	***P*-value**	**FDR *P*-value**	**Chromosome**
SRM	Spermidine synthase	1.49	1.01E-06	5.11E-05	chr1
ODC1	Ornithine decarboxylase 1	1.67	0.0008	0.0062	chr2
SMOX	Spermine oxidase	1.5	2.68E-10	2.23E-07	chr20
RRM2	Ribonucleotide reductase M2	1.89	2.99E-06	0.0001	chr2
SAT1	Spermidine/spermine N1-acetyltransferase 1	1.24	0.0237	0.0804	chrX

We also conducted the bioprofiler analysis of the six candidate genes using Ingenuity pathway analysis software. These analyses reveal the unknown effect of SMOX, SMS, and SRM genes in colorectal cancer. Other genes including ODC1 were found to affect colorectal cancer but no causal relationship has been reported so far (Table S6).

## Discussion

In this report, we have attempted to build genome scale metabolic model for colorectal cancer and identified key metabolic pathways altered in a local patient cohort. Further, we found six genes that could be responsible for these metabolic changes and studied their functional relevance in association with colorectal cancer. Finally, we assessed the prognostic significance of these genes.

Integrated models have recently been successfully used to identify driver genes for colorectal cancer (31–33). However, the patient heterogeneity limits the application of this approach in the clinic. Hence a personalized approach would be desirable for developing appropriate therapeutic regimens. We took a patient wise analysis approach where gene expression profile of colorectal cancer tumor samples was matched with their own normal colon tissue. Glutathione (GSH) metabolism is known to be associated with colorectal cancer pathogenesis. Elevated levels of glutathione and the enzymes responsible for its production have been reported in CRC and this is confirmed by our results. This observation reinforces the hypothesis that rapidly growing cancer cells are under oxidative stress and need more glutathione (34, 35). GSH metabolism could be used as an important biomarker for resistance and treatment response (36). Significant involvement of glutathione metabolism in our patient cohort could be reflective of aggressive tumors since most of the patients were from advanced stages. GSH metabolism could therefore be utilized as a prognostic marker in CRC patients. In addition, polyamine metabolism was identified as elevated in colorectal cancer patients, and it has been associated with tumor growth as well as immune cell function (37). It has been shown that down-regulating one of the key driver of polyamine metabolism, IDO1, could improve the response rate of immunotherapy in cancer (38). Moreover, Hayes et al. reported that by using a “polyamine-blocking therapy,” they could reverse immunosuppression in tumor microenvironment in mice breast cancer model (39). Therefore, the identified polyamine metabolism related genes, especially ODC1 and SMOX, should be tested in *in vitro* and *in vivo* models in future studies since they could be promising gene target for CRC treatment.

ODC1 gene polymorphism has been reported to reduce the risk of adenoma recurrence by suppressing synthesis of colonic mucosal polyamines (40). ODC1 protein has been used as a biomarker for measuring the efficacy of colecoxib in the treatment of colorectal cancer. It has also been involved in familial adenomatous polyposis as a manifestation of APC gene mutation (41). However, there is no known correlation of altered ODC1 gene expression levels with CRC.

Our results thus provide added evidence to support the involvement of ODC1 gene in colorectal cancer progression. ODC1, SAT1, SMOX, and SRM genes are known to decrease cell proliferation in several cancer cell lines (42–46). ODC1 and RRM2 have also been implicated in increased cell proliferation in different conditions (47–50). However, our study is the first to report the putative involvement of SAT1, SMOX, SRM, and RR M2 in colorectal cancer. Sulindac has been reported to induce SAT1 gene mediated chemo preventive affect in colorectal cancer in a COX independent mechanism (51). SMS gene is completely novel in this study with no known prior association with any type of cancer.

The importance of proline metabolism in helping cancer cells to grow is being revealed through this report (52). Our study lends credibility to the putative role of proline metabolism in cancer progression. However, current literature is scarce and more definitive studies are needed to substantiate the role of proline metabolism especially in colorectal cancer. Arginine deprivation therapy has been proposed to be a promising way to deal with cancer at metabolic level (53).

Our validation results of expression of six genes in colorectal patients and cell lines corroborate an interesting aspect of cancer—heterogeneity. While some patients show expected pattern of gene expression, there are others who exhibit a reverse pattern. These results suggest and support the notion of personalized medicine especially in colorectal cancer (54). More studies are needed to generate evidence in favor of customizing dosage and therapeutic agents in patients based on their integrated metabolic profile. Integrated evidence from transcriptomics and metabolomics could be more promising in terms of accuracy and specificity in the clinic (55, 56). Evidence is mounting in favor of using multiomics approach toward finding biomarkers and therapeutic targets especially in cancer (57, 58).

## Data Availability

The datasets generated for this study can be found in the GEO database: GSE50421/https://www.ncbi.nlm.nih.gov/geo/query/acc.cgi?acc=GSE50421, GEO database: GSE77434/https://www.ncbi.nlm.nih.gov/geo/query/acc.cgi?acc=GSE77434.

## Ethics Statement

The studies involving human participants were reviewed and approved by King Abdullah International Medical Research Center. The patients/participants provided their written informed consent to participate in this study.

## Author Contributions

CZ analyzed the data and wrote the paper. MAl did the qRT-PCR experiments. MAr analyzed the data. XL helped in data analysis. AM conceived the idea, designed the analyses, and wrote the paper. MAA conceived the idea, generated and analyzed patient gene expression data, and wrote the paper. All authors read the manuscript and approved its contents.

### Conflict of Interest Statement

The authors declare that the research was conducted in the absence of any commercial or financial relationships that could be construed as a potential conflict of interest.
